# Progesterone through Progesterone Receptor B Isoform Promotes Rodent Embryonic Oligodendrogenesis

**DOI:** 10.3390/cells9040960

**Published:** 2020-04-14

**Authors:** Juan Carlos González-Orozco, Aylin Del Moral-Morales, Ignacio Camacho-Arroyo

**Affiliations:** Unidad de Investigación en Reproducción Humana, Instituto Nacional de Perinatología-Facultad de Química, Universidad Nacional Autónoma de México (UNAM), Ciudad de Mexico 04510, Mexico; g.orozco221@gmail.com (J.C.G.-O.); aylindmm@gmail.com (A.D.M.-M.)

**Keywords:** oligodendrocyte progenitor cells, oligodendrogenesis, myelination, progesterone, progesterone receptor

## Abstract

Oligodendrocytes are the myelinating cells of the central nervous system (CNS). These cells arise during the embryonic development by the specification of the neural stem cells to oligodendroglial progenitor cells (OPC); newly formed OPC proliferate, migrate, differentiate, and mature to myelinating oligodendrocytes in the perinatal period. It is known that progesterone promotes the proliferation and differentiation of OPC in early postnatal life through the activation of the intracellular progesterone receptor (PR). Progesterone supports nerve myelination after spinal cord injury in adults. However, the role of progesterone in embryonic OPC differentiation as well as the specific PR isoform involved in progesterone actions in these cells is unknown. By using primary cultures obtained from the embryonic mouse spinal cord, we showed that embryonic OPC expresses both PR-A and PR-B isoforms. We found that progesterone increases the proliferation, differentiation, and myelination potential of embryonic OPC through its PR by upregulating the expression of oligodendroglial genes such as neuron/glia antigen 2 (NG2), sex determining region Y-box9 (SOX9), myelin basic protein (MBP), 2′,3′-cyclic-nucleotide 3′-phosphodiesterase (CNP1), and NK6 homeobox 1 (NKX 6.1). These effects are likely mediated by PR-B, as they are blocked by the silencing of this isoform. The results suggest that progesterone contributes to the process of oligodendrogenesis during prenatal life through specific activation of PR-B.

## 1. Introduction

Central nervous system (CNS) myelination is a process that begins in late stages of mammalian prenatal life and extends to adulthood [1,2]; however, the oligodendrocytes responsible of this process arise earlier in embryonic neurodevelopment by the specification of neural stem cells (NSC) into oligodendroglial progenitor cells (OPC) after the neurogenic phase [3], which occurs approximately on day 12.5 of the mouse embryonic development and between gestational weeks 16–18 in the human [4,5].

The first site of OPC specification during mammalian neurodevelopment has been identified in the ventral region of the neural tube, in the prospective cervical portion of the spinal cord [4,6,7]. In this region, after the first wave of generation of the motoneurons of the spinal cord, the morphogen sonic hedgehog (Shh) promotes the induction of the NSC into OPC by upregulating the expression of a transcriptional program associated to the oligodendroglial lineage [6,8]; among the upregulated genes in this early specification stage are the transcription factors sex determining region Y-box9/10 (Sox9/10), oligodendrocyte transcription factor 1/2 (Olig1/2), NK6 homeobox 1/2 (Nkx 6.1/6.2) and the receptor α for the platelet-derived growth factor (PDGFRa) [8,9,10]. Then, newly specified OPC actively proliferate and migrate to establish a homogenous distribution [4,7,11].

The proliferation and migratory phenotype of early OPC is maintained by the platelet-derived growth factor (PDGF) and the fibroblast growth factor 2 (FGF2) [7,12], whereas mitotic arrest and subsequent oligodendrocyte differentiation is promoted by several elements such as thyroid hormones [13,14], the ciliary neurotrophic factor (CNTF), the extracellular matrix component laminin α2 (merosin), and the presence of neuronal activity [15,16,17]; final maturation to myelinating oligodendrocytes is induced by the upregulation of promyelinating genes such as the myelin basic protein (MBP), myelin proteolipid protein (PLP), and 2′,3′-cyclic-nucleotide 3′-phosphodiesterase (CNP1) [17,18].

Along with these elements, some studies have shown that progesterone also has a role in developmental oligodendrogenesis and the myelination process [19]; in fact, because the main role of this hormone lies in the maintenance of pregnancy, progesterone is constitutively present in the maternal and embryo-fetal circulation throughout development [20], and continues to be synthesized in the CNS of both females and males after birth [21]. In the adult, it regulates different functions such as neuroprotection, neurogenesis, neuronal plasticity, sexual behavior, mood, learning, and memory [22,23].

Progesterone exerts its effects mainly through two pathways: a directly genomic pathway and a non-genomic pathway. Through the genomic pathway, it interacts with its intracellular receptor (PR), a transcription factor that, once activated, binds to specific DNA sequences named progesterone response elements (PRE) that are mainly located in gene promoter regions, thus regulating their expression [24,25]. Meanwhile, the non-genomic pathway is related to rapid cellular responses triggered by the activation of different G protein-coupled membrane receptors to progesterone (mPRs) or PR ligand-independent activation [26,27].

There are two PR isoforms, PR-A and PR-B, which are encoded in the same gene but from different transcription start sites [28], which makes the PR-A isoform an N-terminally truncated form of the complete PR-B isoform [29,30]. In the brain and the spinal cord, they present a different expression pattern, regulation, and function [31,32,33,34,35]. Generally, given that the PR-B is a more potent transcription activator than the PR-A, the former is the positive regulator of the genomic effects of progesterone, whereas PR-A is mainly a transcriptional repressor [36,37,38].

The effects of progesterone on developmental oligodendrogenesis and myelination have been documented in cerebellar organotypic cultures obtained from postnatal rats and mice. It was demonstrated that the addition of progesterone to the culture medium immediately increases OPC proliferation, and after 1 week it increases the number of differentiated oligodendrocytes and its potential of nerve myelination, as seen by an increasing in MBP expression, effects that were found to be mediated by a genomic mechanism of action because the use of the PR antagonist mifepristone blocked them [39,40]. In addition, it was reported that OPC from the brain of newborn rats actively synthetizes and metabolizes progesterone during its proliferative phase prior to its differentiation into oligodendrocytes, suggesting that autocrine and paracrine actions of progesterone contribute to oligodendrocyte development and nerve myelination [41].

Moreover, progesterone also has effects on CNS remyelination in adulthood because it increases the number of differentiated oligodendrocytes and upregulates the expression of genes involved in myelin repair in adult rats with spinal cord injury. Therefore, progesterone has been proposed to treat demyelinating lesions [42,43].

Despite these studies, it is unknown if progesterone also promotes differentiation of OPC during embryonic life, as well as the specific PR isoform involved in progesterone actions in these glial cells, which is relevant to understand the mechanisms underlying the developmental oligodendrogenesis and thus elaborate therapeutic strategies to treat demyelinating lesions in postnatal life. In this work, we studied the effects of progesterone and the role of PR in the proliferation, differentiation, and myelination potential of OPC, as well the effects of progesterone on the regulation of genes associated with developmental oligodendrogenesis by using isolated OPC from the embryonic mouse spinal cord. Finally, we studied the specific role of PR-B in the embryonic OPC.

## 2. Materials and Methods

### 2.1. Animals

Female CD-1 mice with 14.5 days of pregnancy were used in this study. For this purpose, female mice purchased from the animal facility of Instituto de Investigaciones Biomédicas (UNAM, México) were mated with expert male mice for 12 h, and then maintained in individual cages with free access to food and water until the day of use. Day 0 of pregnancy was defined as the day on which the vaginal plug was observed. Animals were euthanized by cervical dislocation and embryos were extracted and transferred to a cold sterile phosphate-buffered saline (PBS) solution. All animals used in this study were handled following the bioethical guidelines approved by the internal committee for the care and use of laboratory animals (CICUAL) of Facultad de Química (UNAM, México).

### 2.2. OPC Culture

The cervical segments of the spinal cord of E14.5 mouse embryos were dissected and disaggregated in 0.1% trypsin (ThermoFisher Scientific, Waltham, MA, USA) during 20 min at 37 °C. Cellular suspension was centrifuged at 1000× *g* for 3 min and then resuspended in DMEM/F-12 (Dulbecco’s Modified Eagle Medium/Nutrient Mixture F-12) with HEPES buffer, no phenol red culture medium (ThermoFisher Scientific) supplemented with B-27 (ThermoFisher Scientific). Then, 10 μL per 1 mL of disaggregated cells were mixed with 10 μL of trypan blue (ThermoFisher Scientific) and manually counted on a hemocytometer. Approximately 5 × 10⁵ cells were plated per well in 12-well plates, with the addition of FGF2 (20 ng/mL) and the epidermal growth factor (EGF; 20 ng/mL) (Peprotech, Rocky Hill, NJ, USA), which were added every 2 days. After 1 week, the formation of cell spheroid aggregates (neurospheres) was observed. Then, neurospheres were disaggregated with 0.025% trypsin during 10 min at 37 °C; cells were counted as described before and 1.5 × 10⁵ cells were plated under the same conditions; however, from the second day on culture, the growth factor PDGF (10 ng/mL) (Peprotech) was added in replacement of EGF. OPC selective expansion was obtained by platting 8 × 10⁴ cells (derived from the disintegration of second passage neurospheres) per well in 12-well plates pretreated with 0.01% poly-L-lysine (Merck, Kenilworth, NJ, USA) with the addition of the growth factors FGF2 (10 ng/mL) and PDGF (10 ng/mL). Approximately 1 week later, cell confluence was observed; then, OPC were detached with 0.025% trypsin during 10 min at 37 °C and subcultured under the same conditions for approximately 1 week. Cells from the 3rd to 10th passage were used to perform the experiments. To perform the cell proliferation and differentiation experiments, the following pharmacological agents were used: triiodo-L-thyronine (T3; Sigma-Aldrich, St. Louis, MO, USA), progesterone (P4; Sigma-Aldrich), RU486 (mifepristone; Sigma-Aldrich), R5020 (promegestone; PerkinElmer Inc., Waltham, MA, USA), and Org OD 02-0 (10-ethenyl-19-norprogesterone; Sigma-Aldrich).

### 2.3. Immunofluorescence

Cells were fixed using 4% paraformaldehyde solution (Sigma-Aldrich) at room temperature for 20 min followed by three washes in PBS. Then, cells were permeabilized and blocked in a PBS solution with 1% bovine serum albumin (BSA; In Vitro, MEX), 1% glycine (Sigma-Aldrich), and 0.2% Triton X-100 (Sigma-Aldrich) at room temperature for 30 min. Cells were incubated at 4 °C for 12 h with primary antibodies at the following dilutions: 1:500 mouse anti- sex determining region Y-box2 (Sox2) (Santa Cruz, Dallas, TX, USA; sc-365964), 1:200 mouse anti-neuron/glia antigen 2 (NG2) (Millipore, Burlington, MA, USA; mab5384), 1:300 mouse anti-oligodendrocyte marker O4 (Millipore; mab345), 1:500 mouse anti-myelin basic protein (MBP) (Millipore; 05-675), 1:100 rabbit anti-microtubule-associated protein 2 (MAP2) (Invitrogen, Waltham, MA, USA; pa5-17646), 1:200 rabbit anti-Ki67 antigen (Millipore; ab9260), and 1:500 rabbit anti-glial fibrillary acidic protein (GFAP) (Abcam, Cambridge, UK; ab7260). Cells were rinsed three times in PBS and then incubated with secondary antibodies goat anti-mouse Alexa Fluor 488 or goat anti-rabbit Alexa Fluor 568, both at dilution 1:500 (ThermoFisher Scientific) at room temperature for 60 min. Nuclei were stained for 5 min with 1 mg/mL Hoechst 33,342 solution (ThermoFisher Scientific). Finally, cells were coverslipped with fluorescence mounting medium (Polysciences Inc., Warrington, PA, USA) and visualized in an Olympus Bx43 microscope (JPN). All immunofluorescence images were captured with a 400X magnification from three random fields per coverslip in each independent experiment. All images were captured under the same gain, exposure time and light intensity conditions. The percentage of immunopositive cells was calculated considering the nuclei stained with Hoechst as the total (100%) number of cells using the “Cell counter” plugin in the ImageJ software ver. 1.51 (NIH, Bethesda, MD, USA). Fluorescence density was measured as “Integrated density” from the Analyze menu of ImageJ, whereas oligodendrocyte branching was measured as “Area” from the same menu options.

### 2.4. RNA Isolation and PCR Experiments

Total RNA was extracted using TRIzol LS reagent (ThermoFisher Scientific) following the manufacturer’s instructions. One microgram of total RNA was used to synthesize the first strand of complementary DNA using the Moloney Murine Leukemia Virus (M-MLV) reverse transcriptase (TermoFisher Scientific) following the manufacturer’s protocol. Two microliters from the previous reaction was used to perform RT-PCR or RT-qPCR experiments using the Platinum Taq DNA Polymerase (ThermoFisher Scientific) and the FastStart DNA Master SYBR Green I reagent kit (Roche, Basel, Switzerland), respectively. RT-PCR results were visualized in a 1.5% agarose (Sigma-Aldrich) gel electrophoresis. RT-qPCR experiments were performed on a LightCycler 1.5 (Roche) and relative expression levels were calculated by the ∆Ct method. The used primers were: NG2: FW 5′-GCCCGTGCCCTCAGC-3′ RV 5′-CAAGTCTGACCTGGAGGCAT-3′; PDGFRa: FW 5′-GGAAGAGGATGACTCTGCCAT-3′ RV 5′-CGAAGCCTTTCTCGTGGACA-3′; OLIG1: FW 5′-GCAGCCACCTATCTCCTCAT-3′ RV 5′-GTGGCAATCTTGGAGAGCTT-3′; SOX9: FW 5′-GAGCTGGAAGTCGGAGAGC-3′ RV 5′-CTCTCGTTCAGCAGCCTCCA-3′; SOX10: FW 5′-AGCCCAGGTGAAGACAGAGA-3′ RV 5′-AGTCAAACTGGGGTCGTGAG-3′; MBP: FW 5′-TCACAGAAGAGACCCTCACA-3′ RV 5′-GCCGTAGTGGGTAGTTCTTG-3′; CNP1: FW 5′-TCCACGAGTGCAAGACGCTATTCA-3′ RV 5′-TGTAAGCATCAGCGGACACCATCT-3′; NKX 6.1: FW 5′-CTTCTGGCCCGGAGTGATG-3′ RV 5′-GGGTCTGGTGTGTTTTCTCTTC-3′; NKX 6.2: FW 5′-CTTCTGGCCCGGAGTGATG-3′ RV 5′-CGGTTGTATTCGTCATCGTC-3′; PR-B: FW 5′-ACCTGAGGCCAGATTCAGAAG-3′ RV 5′-GTATCCCCCACATGCACGTAT-3′ and 18S ribosomal RNA: FW 5′-AGTGAAACTGCAATGGCTC-3′ RV 5′-CTGACCGGGTTGGTTTTGAT-3′.

### 2.5. Analysis of Potential Progesterone Response Elements

The promoter sequences and transcription start sites were obtained from the Eukaryotic Promoter Database (EPD) (https://epd.epfl.ch//index.php); potential binding sites for PR were searched using the algorithms contained in JASPAR [44], TRANSFAC [45], and NUBIscan [46] databases. The binding sites predicted by two or more databases with a matrix similarity score higher than 0.8 were established as potential PRE.

### 2.6. Western Blot

OPC were detached from culture plates using PBS-EDTA (ethylenediaminetetraacetic acid; Sigma-Aldrich) and cell scraper (Corning, New York, NY, USA). Cells were centrifuged and obtained pellets were lysed with radioimmunoprecipitation assay (RIPA) buffer and protease inhibitors (1 mM EDTA, 2 µg/mL leupeptin, 2 µg/mL aprotinin, 1 mM phenylmethanesulfonyl fluoride (PMSF); Sigma-Aldrich). Total protein was extracted by centrifugation at 20,817× *g* at 4 °C for 15 min. The protein concentration of each sample was measured using the Pierce 660 nm Protein Assay reagent (ThermoFisher Scientific) and NanoDrop-2000 Spectrophotometer (ThermoFisher Scientific). A total of 100 µg of total protein was separated on 10% SDS-PAGE at 80 V for 2 h and then transferred to a polyvinylidene fluoride (PVDF) membrane (Merck) for 1 h at 25 V in semi-dry conditions at room temperature. Membranes were blocked overnight with 5% BSA (In Vitro) and then incubated at 4 °C with primary antibodies mouse anti-PR in dilution 1:300 (Abcam; ab58568) or rabbit anti-α tubulin in dilution 1:1000 (Santa Cruz; sc-5286) for 48 h. Blots were incubated with goat anti-rabbit secondary antibody conjugated to horseradish peroxidase (HRP) in dilution 1:4500 (Santa Cruz; sc-2004) or mouse IgG kappa binding protein (mIgGκ BP) conjugated to HRP in dilution 1:5000 (Santa Cruz; sc516102) at room temperature for 45 min. Immunoreactive species were detected by chemiluminescence exposing blots to Kodak Biomax Light Film (Sigma-Aldrich) after incubation with Super Signal West Femto Maximum Sensitivity Substrate reagent (ThermoFisher Scientific). Blot images were captured with a digital camera (Sony, Tokyo, Japan); the densitometric analysis was performed using the ImageJ software ver. 1.51 (NIH).

### 2.7. siRNA-Mediated PR-B Silencing

Cultured OPC at 60–80% confluence were subjected to siRNA (small interfering RNA) transfection. The day of transfection, the cell medium was replaced with DMEM/F-12, HEPES phenol-red free without B27, antibiotics, and growth factors. Then, the cells were transfected with a PR-B siRNA duplex (150 nM) or with a control siRNA (150 nM) without any specific mRNA target sequence (Sigma-Aldrich) using the reagent Lipofectamine RNAiMAX (ThermoFisher Scientific) and following the manufacturer’s instructions. Then, 24 h later, the transfection medium was exchanged with DMEM/F-12 and HEPES phenol-red free supplemented with B27 and growth factors. PR-B silencing was corroborated 48 h after transfection by RT-qPCR. The sequence of the employed mouse PR-B siRNA duplex was sense 5′-AUGACUGAGCUGCAGGCAA-3′, antisense 5′-UUGCCUGCAGCUCAGUCAU-3′.

### 2.8. Statistical Analysis

All data were analyzed and plotted with the GraphPad Prism 5.0 software (GraphPad, San Diego, CA, USA). Plotted data are representative of three independent experiments (i.e., from three different and independent cell cultures). Statistical analysis between comparable groups was performed using a one-way ANOVA with a Bonferroni post-test. RT-qPCR, Western blot, and siRNA silencing data were analyzed with a two-tail unpaired Student’s *t*-test. Values of *p* < 0.05 were considered statistically significant.

## 3. Results

### 3.1. Oligodendrocyte Progenitor Cultures Derived from the Mouse Embryonic Spinal Cord

**Figure 1:**
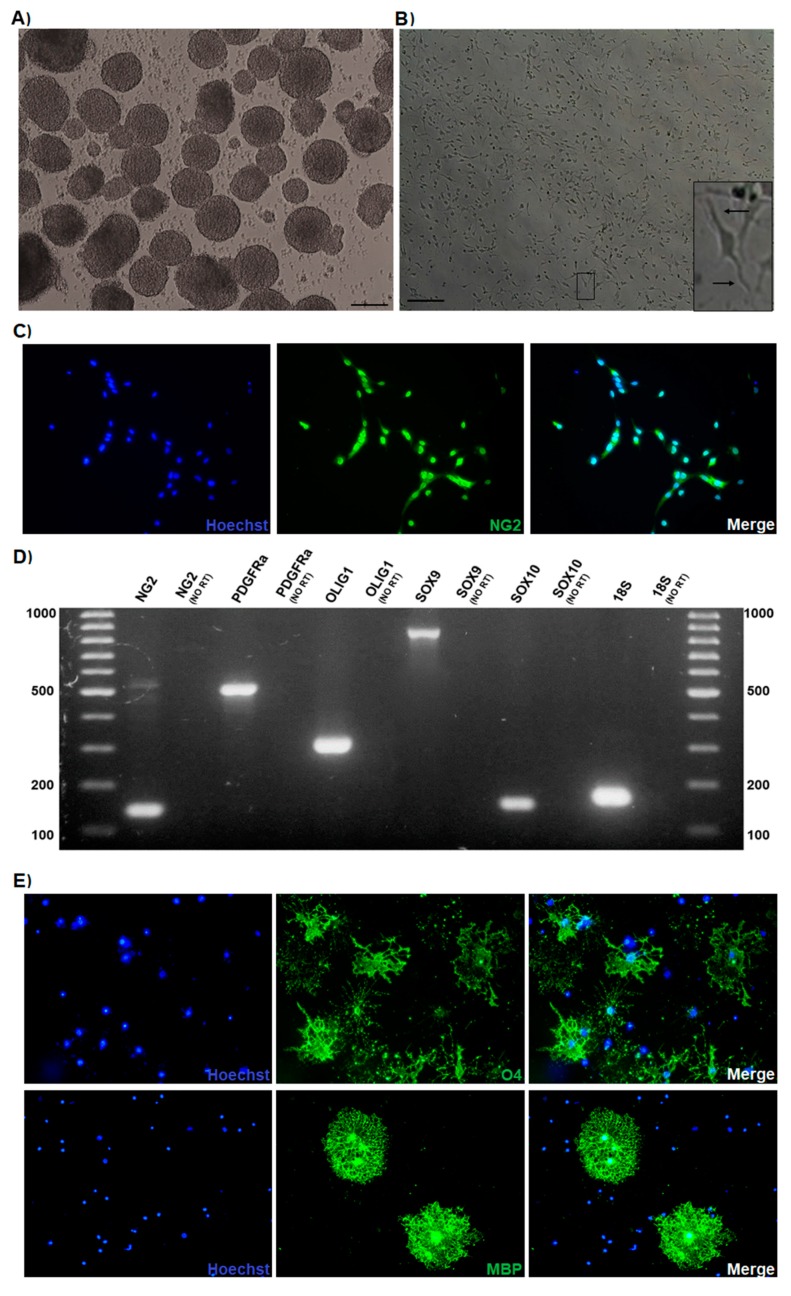
Oligodendroglial progenitor cells (OPC) cultures derived from the spinal cord of E14.5 mouse embryos. (**A**) Primary neurosphere cultures generated from the mouse embryonic spinal cord. (**B**) Selective OPC expansion from the neurospheres. The arrows in the zoomed area shows the bipolar processes. Scale bars: 100 μm. (**C**) Immunodetection of the typical OPC marker NG2 in the cultured cells. Nuclei stained with Hoechst are shown in blue, anti-NG2 primary antibody is shown in green, and merge panel shows the combination of blue and green colors. (**D**) The expression of the oligodendrogial genes NG2 (143 bp), PDGFRa (524 bp), OLIG1 (309 bp), SOX9 (820 bp), and SOX10 (146 bp) was detected in the cultured cells by RT-PCR. (**E**) The OPC were cultured without growth factors and treated for 3 days with T3 hormone (40 ng/mL); differentiation was evaluated by immunodetection of the oligodendrocyte marker O4 and the myelin marker myelin basic protein (MBP). All immunofluorescence images were captured with 400× magnification.

The cervical segments of the spinal cord of E14.5 mouse embryos (Figure S1A) were isolated to prepare primary neurosphere cultures (Figure 1A), as previously described [47]. The obtained neurospheres were mainly composed of cells positive for the stem cells marker Sox2 (Figure S1B,C). Then, OPC were selectively expanded by disaggregation of the neurospheres and plating the cells in adherent conditions supplemented with the growth factors FGF2 and PDGF (each one at 10 ng/mL); the effectiveness of obtaining cells from the oligodendroglial lineage by following this procedure has been previously demonstrated [48,49]. Highly proliferative cells with bipolar morphology were observed shortly after plating (Figure 1B and Figure S1D), which is the morphology associated with the OPC phenotype [49]. To confirm the oligodendroglial lineage, cells were immunostained with the typical OPC marker NG2 (Figure 1C); as shown, most of the cells were NG2 positive, and the signal was mainly detected in their nuclei and processes. Furthermore, the expression of oligodendroglial lineage genes was detected in OPC by RT-PCR experiments (Figure 1D). To determine whether the cultured OPC responded to well-known differentiation stimuli, cells were cultured without growth factors and treated for 3 days with the thyroid hormone T3 (40 ng/mL). After treatment, morphological changes were observed; additionally, the cells were immunostained positively for O4, a typical marker of differentiated oligodendrocytes. Some MBP (myelin marker)-positive cells were spotted (Figure 1E). Additionally, immunofluorescence to detect MAP2- or GFAP-positive cells after culturing the cells for 3 days without growth factors showed the presence of a low number of neurons or astrocytes, respectively (Figure S1E). With these results, the efficacy to obtain a large amount of highly pure functional OPC from the spinal cord of E14.5 mouse embryos was demonstrated by following the described procedure.

### 3.2. Progesterone Promotes the Proliferation and Differentiation of Mouse Embryonic OPC and Increases Its Potential of Myelination through the PR

**Figure 2:**
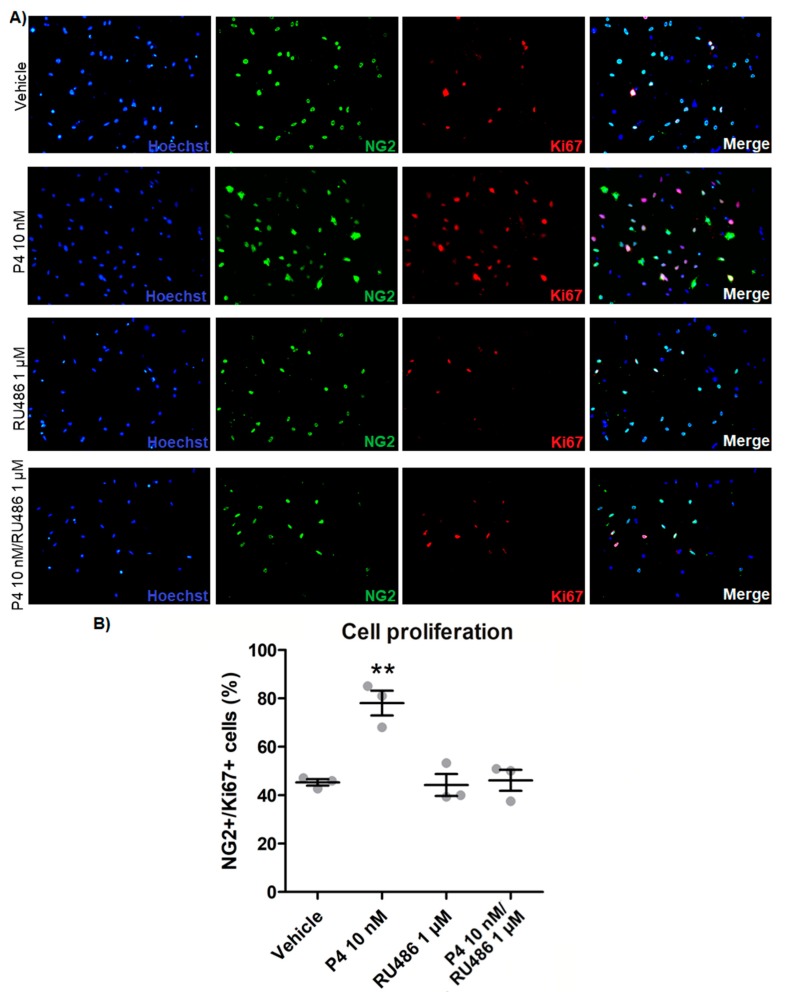
Progesterone increases embryonic OPC proliferation. (**A**) NG2/Ki67 immunostaining in OPC cultured with growth factors and treated with progesterone (P4; 10 nM), RU486 (1 µM), P4 (10 nM) and RU486 (1 µM), and vehicle (DMSO 0.01%), for 3 days. (**B**) Graph derived from the percentage of NG2/Ki67-positive cells observed in the immunofluorescence experiments. The percentage of NG2+/Ki67+ cells was determined as a percentage of the total number of cells stained with Hoechst. Results are expressed as the mean ± standard error of the mean (S.E.M.); *n* = 3; ** *p* < 0.01 vs. the rest of the groups.

Once the protocol for obtaining OPC from the mouse embryonic cord was established, the effects of progesterone on cell proliferation were evaluated. For this purpose, OPC were cultured with the addition of growth factors (FGF2 and PDGF at 10 ng/mL each one) and treated for 3 days with progesterone (10 nM), the competitive PR antagonist RU486 (1 μM), or progesterone and RU486. As shown in Figure 2, progesterone increased the percentage of Ki67/NG2-positive cells as compared with the vehicle control, whereas the treatment with only RU486 did not induce any change. RU486 blocked progesterone effect, suggesting that progesterone promotes OPC proliferation through its PR. To further confirm this statement, cell proliferation was assessed treating the OPC with the PR agonist R5020 (10 nM), or with the mPRs-specific agonist Org OD 02-0 (100 nM). As observed with progesterone, the agonist R5020 increased the number of proliferating Ki67/NG2 cells, whereas the mPRs agonist did not increase it (Figure S2). 

**Figure 3:**
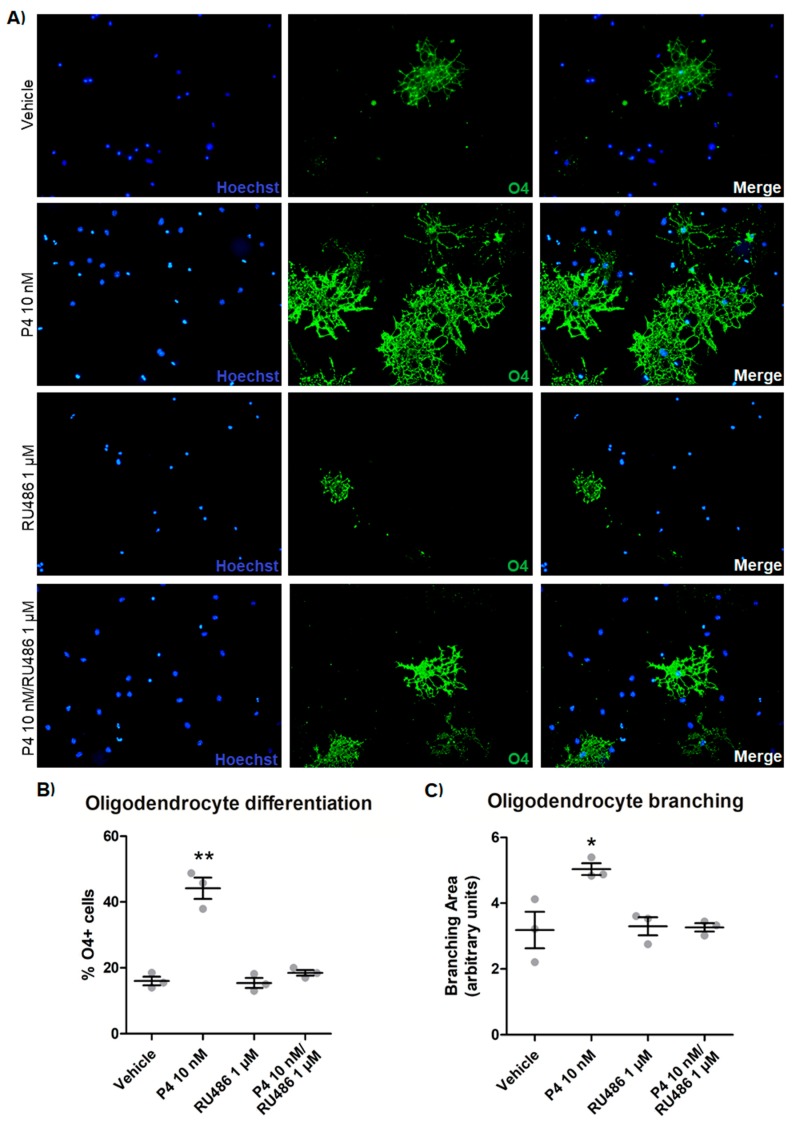
Progesterone promotes oligodendrocyte differentiation. (**A**) O4 immunofluorescence in OPC cultured without growth factors and treated for 3 days with P4 (10 nM), RU486 (1 µM), P4 (10 nM) and RU486 (1 µM), and vehicle (DMSO 0.01%). (**B**) Percentage of O4-positive cells observed in the immunofluorescence experiments. The percentage of O4+ cells was determined as a percentage of the total number of cells stained with Hoechst. (**C**) Cellular branching measured in O4-positive cells. Results are expressed as the mean ± S.E.M. *n* = 3; * *p* < 0.05 and ** *p* < 0.01 vs. the rest of the groups.

Progesterone also promoted the differentiation of OPC that were cultured without growth factors and treated for 3 days, as seen by a striking increase in the number of O4-positive cells in immunofluorescence preparations (Figure 3A,B). With this approach, it was also observed that progesterone significantly increased the branching area of the differentiated oligodendrocytes (Figure 3C), and all these effects were mediated by the PR because the treatments with RU486 blocked them (Figure 3). Moreover, R5020 induced similar results as compared with progesterone, whereas Org OD 02-0 did not induce any effect (Figure S3). 

**Figure 4:**
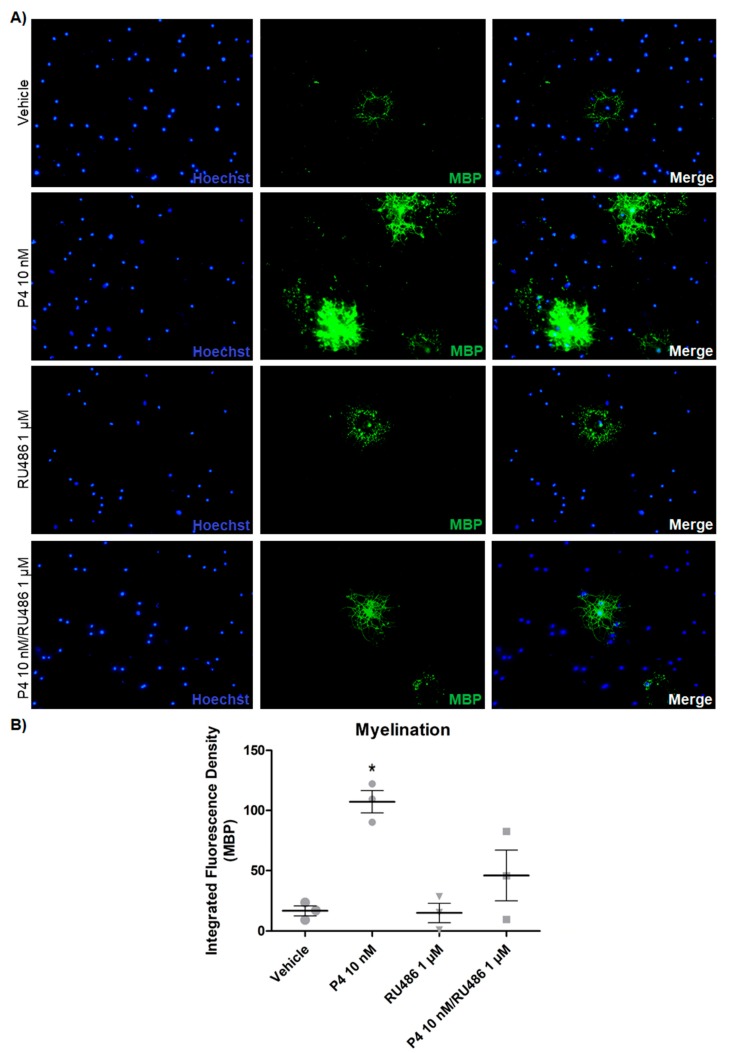
Progesterone increases the potential of myelination of differentiated OPC. (**A**) MBP immunofluorescence in OPC cultured without growth factors and treated for 3 days with P4 (10 nM), RU486 (1 µM), P4 (10 nM) and RU486 (1 µM), and vehicle (DMSO 0.01%). (**B**) MBP expression measured as a fluorescence density. Results are expressed as the mean ± S.E.M. *n* = 3; * *p* < 0.05 vs. vehicle and RU486.

Regarding the potential of myelination, progesterone increased the expression of the myelin marker MBP in the differentiated OPC, and RU486 blocked this effect (Figure 4). Moreover, the PR agonist R5020 exerted similar effects to those of progesterone. In contrast, Org OD 02-0 did not produce any significant change in MBP expression (Figure S4). These results suggest that progesterone through a genomic mechanism of action regulates the proliferation and differentiation of mouse embryonic OPC, also increasing their myelinating function. 

### 3.3. Progesterone Upregulates the Expression of Oligodendroglial Genes

**Figure 5:**
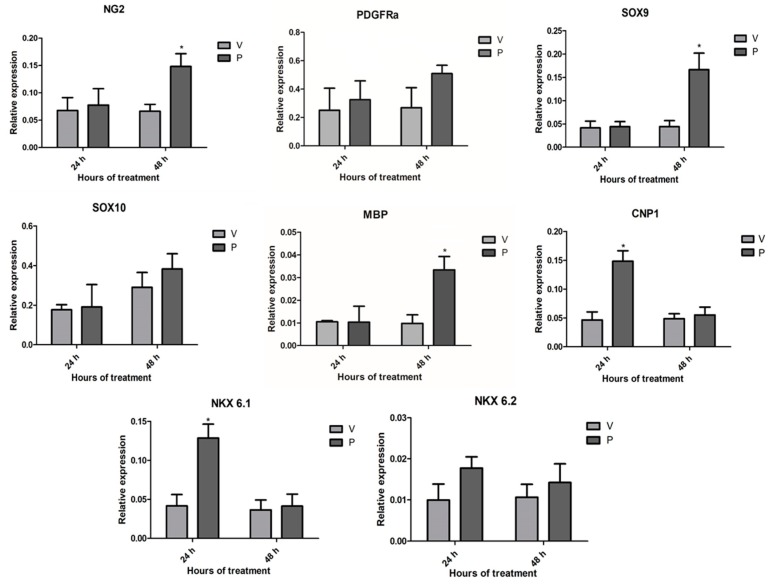
Progesterone upregulates the expression of oligodendroglial genes. The graphs show the relative gene expression (normalized to the 18S gene expression) of the NG2, PDFGR, SOX9, SOX10, MBP, 2′,3′-cyclic-nucleotide 3′-phosphodiesterase (CNP1), NKX 6.1, and NKX 6.2 genes obtained by RT-qPCR after treating OPC with P4 (10 nM) and vehicle (DMSO 0.01%) for 24 and 48 h. Results are expressed as the mean ± S.E.M. *n* = 3; * *p* < 0.05 vs. vehicle.

Considering that progesterone promoted the proliferation and differentiation of the cultured OPC through the specific activation of the PR (i.e., by a genomic mechanism of action), the effects of progesterone on the regulation of the expression of various genes associated with the embryonic oligodendrogenesis were studied. After 24 h of exposure, progesterone upregulated the expression of the transcription factor Nkx 6.1, as well as the expression of the myelin marker Cnp1 in the OPC, whereas after 48 h, the upregulated genes by progesterone were NG2; the transcription factor Sox9; and, as expected, the myelin marker MBP. Although it was not observed that progesterone significantly increased the expression of the genes of PDGFRa, Sox10, and Nkx 6.2, an increasing trend was observed in cells exposed to the hormone (Figure 5). The PR works as a transcription factor that binds to DNA sequences known as PRE, which are usually located in promoter regions of target genes. In order to detect potential PRE sequences in the promoter regions of the progesterone-upregulated genes, an in silico analysis was performed. The promoter sequences of the NG2, Sox9, Nkx 6.1, Cnp1, and MBP genes were obtained from the EPD and confirmed by a BLAST (Basic Local Alignment Search Tool) analysis (https://blast.ncbi.nlm.nih.gov/Blast.cgi). By using the algorithms from the JASPAR, TRANSFAC, and NUBIscan databases, it was identified that all genes have potential PRE in their promoter regions (Figure S5). Based on these results, it was confirmed that the effects of progesterone on embryonic OPC were mediated by a genomic mechanism of action, and that in addition, progesterone can differentially regulate the expression of genes associated with oligodendrogenesis throughout embryogenesis.

### 3.4. The Mouse Embryonic OPC Express the PR-A and PR-B Isoforms and the Oligodendrogenic Actions of Progesterone are Mediated by the PR-B

**Figure 6:**
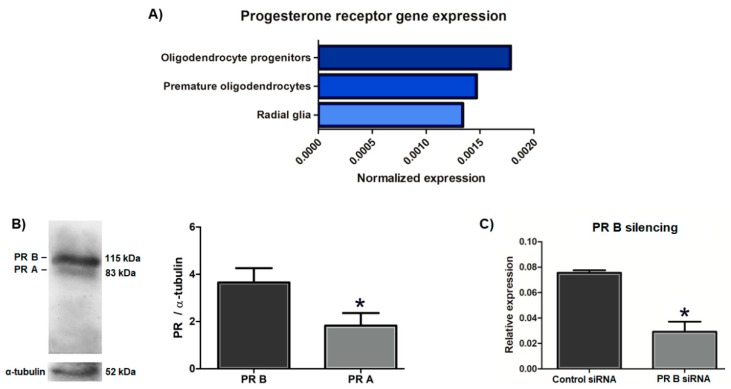
The OPC from the mouse embryonic spinal cord express both progesterone receptor (PR) isoforms. (**A**) PR gene expression analyzed by single cell RNA-seq in radial glial cells, oligodendrocyte progenitors, and premature oligodendrocytes from E9.5-E13.5 whole mouse embryos obtained from the “Mouse Organogenesis Cell Atlas” [50]. (**B**) PR-A and PR-B expression detected by Western blot in the cultured OPC derived from the spinal cord of E14.5 mouse embryos. (**C**) PR-B silencing by siRNA transfection in the cultured OPC corroborated by RT-qPCR. Results are expressed as the mean ± S.E.M. *n* = 3; * *p* < 0.05 vs. PR-B; * *p* < 0.05 vs. control siRNA.

Because PR expression in embryonic OPC is not yet reported, we first searched in the “Mouse Organogenesis Cell Atlas” (MOCA), using the “Genes” tool (https://oncoscape.v3.sttrcancer.org/atlas.gs.washington.edu.mouse.rna/landin) [50], if PR expression in mouse embryonic oligodendroglial cells was spotted by single-cell RNA sequencing (RNA-seq) during the assembly of this database. According to this database, radial glial cells, OPC, and premature oligodendrocytes from whole mouse embryos between 9.5 and 13.5 days of gestation express the PR; moreover, the expression of the PR increases when radial glial cells are specified towards OPC, and then decreases when they differentiate into oligodendrocytes (Figure 6A), suggesting a particular role of the PR in OPC. We detected the expression of PR-A and PR-B isoforms in the cultured embryonic OPC by Western blot, noticing that PR-B is expressed in a higher proportion than PR-A (Figure 6B). Given that PR-B is a more potent transcription activator than PR-A, and because it was observed that the effects of progesterone in the proliferation and differentiation of the OPC were mediated by a genomic mechanism, the expression of PR-B was silenced by siRNA transfection in order to study the role of this isoform in the mouse OPC. PR-B silencing was corroborated by RT-qPCR, observing that the transfection with the PR-B siRNA diminished PR-B expression by more than 60% as compared with the control siRNA (Figure 6C).

**Figure 7:**
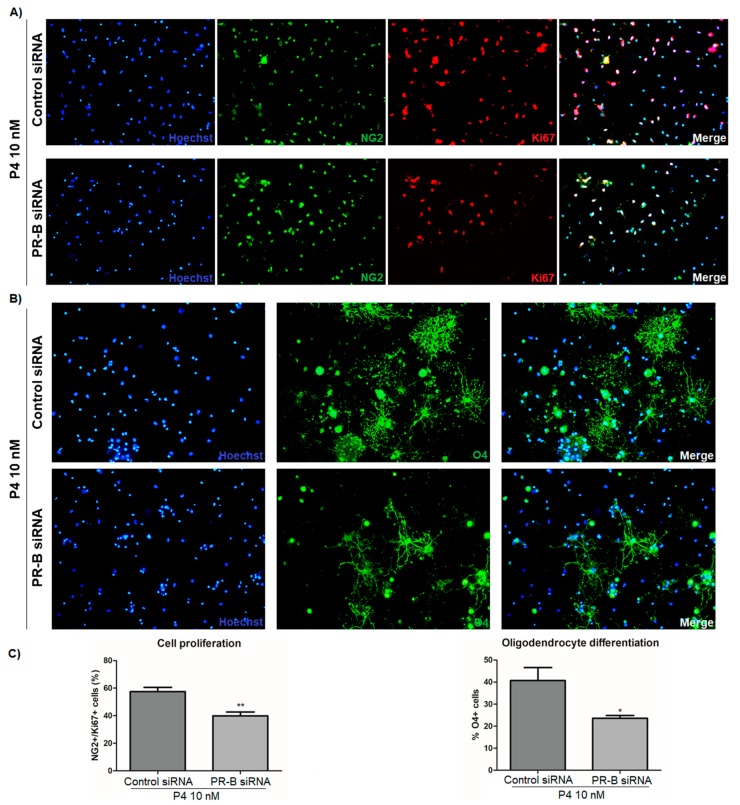
The silencing of PR-B blocks the oligodendrogenic effects of progesterone. (**A**) NG2/Ki67 and (**B**) O4 immunofluorescence in OPC transfected with a control siRNA or with a PR-B siRNA and treated for 3 days with P4 (10 nM). (**C**) Graphs derived from the percentage of NG2/Ki67 and O4-positive cells observed in the immunofluorescence. Results are expressed as the mean ± S.E.M. *n* = 3; * *p* < 0.05 and ** *p* < 0.01 vs. control siRNA.

Twenty-four hours after transfection, the OPC were treated for 3 days with progesterone, and then cell proliferation and differentiation were determined by immunofluorescence. We noticed a significant lower percentage of NG2/Ki67 and O4-positive cells in the PR-B siRNA-transfected cells as compared with the control siRNA-transfected cells (Figure 7). This indicates that the oligodendrogenic effects of progesterone are mediated by PR-B.

## 4. Discussion

Progesterone functions extend beyond the regulation of female reproduction. In fact, because it is synthesized in the CNS of both males and females, it is referred as a neurosteroid and regulates several neural functions including neuroprotection, neuromodulation, neurogenesis, neuronal plasticity, and nerve remyelination [22,23]. Moreover, progesterone also participates in critical development events such as neuronal differentiation, neural circuit organization, brain sex differentiation, and oligodendrogenesis [51].

The effects of progesterone on nerve myelination were first documented in peripheral nerves, where progesterone promotes the remyelination after traumatic injury [52]. These observations were later expanded to the CNS. It has been reported that progesterone has neuroprotective and promyelinating effects on adult individuals with spinal cord injury [42,43], leading to the proposal of the therapeutic use of progesterone for the treatment of demyelinating lesions [43,53]. Regarding the developmental oligodendrogenesis, it is documented that OPC from the brain of newborn rats synthesizes progesterone [41], and that this hormone also promotes, through the activation of its PR, the proliferation and differentiation of the OPC, while also increasing the developmental nerve myelination, as has been observed in the cerebellum of postnatal rodents [39,40]. Nonetheless, it is unknown if progesterone also promotes the proliferation and differentiation of OPC during early oligodendrogenesis in embryonic life, as well as the PR isoform involved in progesterone effects, which is relevant in order to understand the mechanisms that underlie embryonic oligodendrogenesis, and thus develop more efficient therapies to alleviate demyelinating diseases and lesions in postnatal life. Therefore, we studied the effects of progesterone and the role of PR-B isoform in OPC primary cultures derived from the spinal cord of E14.5 mouse embryos.

The developing mouse spinal cord is an accessible embryonic tissue that contains diverse CNS progenitor cells and a reliable study model because the spatial and temporal events underlying the neurogenesis and gliogenesis in this tissue are well defined [54,55]. The specification of the NSC into OPC during mouse neurodevelopment occurs on the embryonic day 12.5 [4]; in addition, it has been reported that NSC isolated from the mouse embryonic spinal cord are more oligogenic than NSC isolated from the developing brain [56]. Thus, we were able to obtain a large amount of highly pure OPC from neurosphere cultures formed primarily of Sox2-positive NSC isolated from the spinal cord of E14.5 mouse embryos. In this study, we showed that progesterone increased the rate of proliferation of the cultured embryonic OPC and promoted its differentiation into oligodendrocytes as previously reported in OPC from the cerebellum of postnatal rodents [39,40]. In addition to this, we observed that progesterone enhanced the potential of myelination of the embryonic OPC, as seen by an increase in MBP protein expression. We were able to observe this latter expression because it has long been known that mouse oligodendrocytes do not need the presence of active neurons to start expressing myelin markers in vitro [57]. The oligodendrogenic effects of progesterone were mediated by the PR because PR agonist R5020 exerted similar effects to those of progesterone, whereas the PR antagonist RU486 blocked them. Likewise, the mPR agonist Org OD 02-0 did not induce any effect. This suggests that progesterone participates in the oligodendrogenesis during prenatal development through a genomic mechanism of action. The genomic effects of progesterone in the OPC were confirmed by evaluating the expression of various genes associated with the specification and maintenance of the oligodendroglial phenotype; specifically, we observed that progesterone increased the expression of Nkx 6.1 and Cnp1 genes 24 h after exposure, whereas the expression of NG2, Sox9, and MBP genes was augmented at 48 h without inducing changes in the expression of Nkx 6.1 and Cnp1, therefore suggesting that progesterone regulates the expression of oligodendroglial genes in a time dependent-manner. Previously, it was reported that progesterone upregulates the expression of the oligodendroglial genes Olig1/2, Nkx 2.2, MBP, myelin proteolipid protein (PLP), and myelin-oligodendrocyte glycoprotein (MOG) in adult rats with complete spinal cord injury, whereas it also increases the number of myelinating oligodendrocytes by promoting the proliferation of the adult remnant OPC and its subsequent differentiation in these same animals [42]. In a similar study, it was also shown that progesterone upregulates the expression of MBP, PLP, Nkx 2.2, and Olig1 in the spinal cord of a murine model of multiple sclerosis. This upregulation was associated with the observed neuroprotective and remyelinating effects of progesterone in these animals [58]. These studies, together with ours, demonstrate that the role of progesterone in the OPC and myelination process during the development or demyelinating lesions is mediated by the upregulation of key oligodendroglial genes. Furthermore, the fact that OPC from the spinal cord of adult individuals are responsive to progesterone, especially during injury, indicates that these cells retain their sensitivity to this hormone since the prenatal state, and it then expands to adulthood.

Previously, the total expression of the PR has been detected in cultured astrocytes and oligodendrocytes isolated from the brain of newborn rats [59], and additionally the expression of the PR-B has been observed by immunohistochemistry in neurons and glial cells of the rat spinal cord with no significant sex differences [60]. In this work, we were able to detect the expression of both PR isoforms in the embryonic OPC by Western blot, with the particular observation that PR-B expression is higher than that of PR-A. Additionally, we examined in the MOCA database [50] the expression of the PR in oligodendroglial cells directly derived from E9.5-E13.5 whole mouse embryos. According to this database, the expression of the PR increases when radial glial cells are specified towards OPC, and then decreases when they differentiate into oligodendrocytes, indicating that PR plays a key role during the OPC stage. Therefore, we demonstrate that early OPC from the embryonic spinal cord of mouse constitutively express PR. Furthermore, because we noticed that the PR-B is more abundant in the OPC, and it is a more potent transcription activator than PR-A, we silenced its expression. Then, we observed that the effects of progesterone on OPC proliferation, differentiation, and myelination potential were significantly blocked, indicating that PR-B is the active mediator of progesterone and its effects on oligodendroglial cells. Nevertheless, we do not discard the possibility that the oligodendrogenic effects of progesterone could be synergistically mediated by both PR isoforms; for example, as transcriptional repressor, PR-A could inhibit the expression of genes that in turn repress the expression of the oligodendroglial genes regulated by PR-B. Thus, this issue deserves to be addressed in future studies.

Our results suggest that progesterone should participate in prenatal oligodendrogenesis. This is plausible because the expression of PR and the expression and activity of the enzymes required for progesterone synthesis have been found from the early embryo ages of the mammalian development in several regions of the developing CNS [61]. Additionally, pregnancy is characterized by an increase in maternal progesterone levels, and there is evidence that progesterone from the maternal circulation enters the embryo/fetal circulation and binds its PR in the developing rat CNS [62]. Remarkably, the levels of progesterone in fetal circulation and in the brain progressively increases throughout pregnancy, especially during late pregnancy [63], coinciding with the time in which the CNS undergoes critical processes such as neural circuits organization and myelination [64].

As mentioned above, the therapeutic use of progesterone to treat CNS injuries and demyelinating lesions has been proposed [43,53], and clinical studies have already been conducted [65]. However, phase 3 clinical trials in patients with traumatic brain injury have failed to replicate the positive outcomes observed in phase 2 trials and in animal models [66]. It is relevant to mention that these clinical trials, as well those previously performed in animal models, did not contemplate the expression or specific function of PR isoforms. Here, we showed that PR-B is predominantly expressed in the embryonic OPC and that it mediates the oligodendrogenic effects of progesterone. These results suggest that the expression and regulation of PR isoforms should determine the oligodendrogenic and promyelinating actions of progesterone in a tissue and developmentally dependent manner.

## Supplementary Materials

The following are available online at https://www.mdpi.com/2073-4409/9/4/960/s1: Figure S1: OPC cultures generated from Sox2-positive neurospheres derived from the spinal cord of E14.5 mouse embryos. Figure S2: Effects of the PR agonist R5020 and the membrane receptors to progesterone (mPR) agonist Org OD 02-0 on OPC proliferation. Figure S3: Effects of the PR agonist R5020 and the mPR agonist Org OD 02-0 on OPC differentiation. Figure S4: Effects of the PR agonist R5020 and the mPR agonist Org OD 02-0 in the potential of myelination. Figure S5: In silico analysis to identify potential progesterone response elements (PRE) sites in the promoter sequences of NG2, SOX9, NKX 6.1, CNP1, and MBP genes.
