# Supplementary material for: Progesterone through Progesterone Receptor B Isoform Promotes Rodent Embryonic Oligodendrogenesis
Source: Cells. 2020 Apr 14;9(4):960. doi: 10.3390/cells9040960 (PMC7226962; doi:10.3390/cells9040960)
Supplement: Supplementary file 1 [file cells-09-00960-s001.pdf]

Progesterone through progesterone receptor B isoform promotes rodent embryonic oligodendrogenesis

-Supplementary figures-

Juan Carlos González-Orozco, Aylin Del Moral-Morales, Ignacio Camacho-Arroyo*.

Unidad de Investigación en Reproducción Humana, Instituto Nacional de Perinatología-Facultad de Química, Universidad Nacional Autónoma de México (UNAM), Ciudad de México, México

*Corresponding author:

Dr. Ignacio Camacho-Arroyo,
Unidad de Investigación en Reproducción Humana,
Instituto Nacional de Perinatología-Facultad de Química,
Universidad Nacional Autónoma de México,
Ciudad de México, México;
Tel: (+52) 55 5520 9900/5622 3732;
E-mail address: camachoarroyo@gmail.com

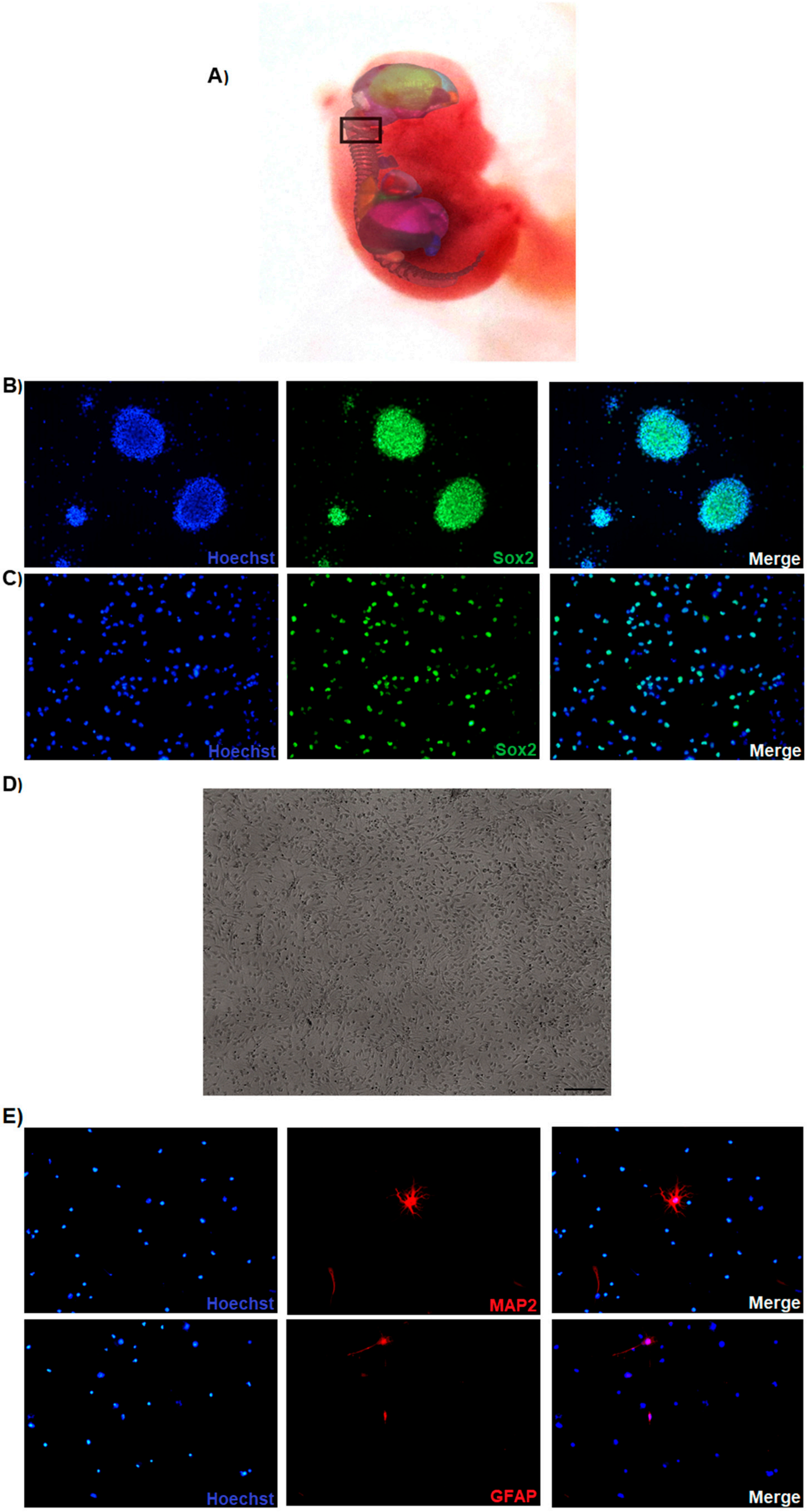

Figure S1. OPC cultures derived from the spinal cord of E14.5 mouse embryos. A) E14.5 mouse embryo; the marked box shows the tissue area that was dissected. B) The primary neurosphere

cultures generated from the mouse embryonic spinal cord express the NSC marker Sox2. C) Sox2 positive cells disaggregated from the neurospheres. D) Highly proliferative cells with bipolar morphology cultured in adherent conditions with FGF2 and PDGF. E) MAP2 and GFAP immunofluorescence show a low number of neurons and astrocytes respectively in the cultures.

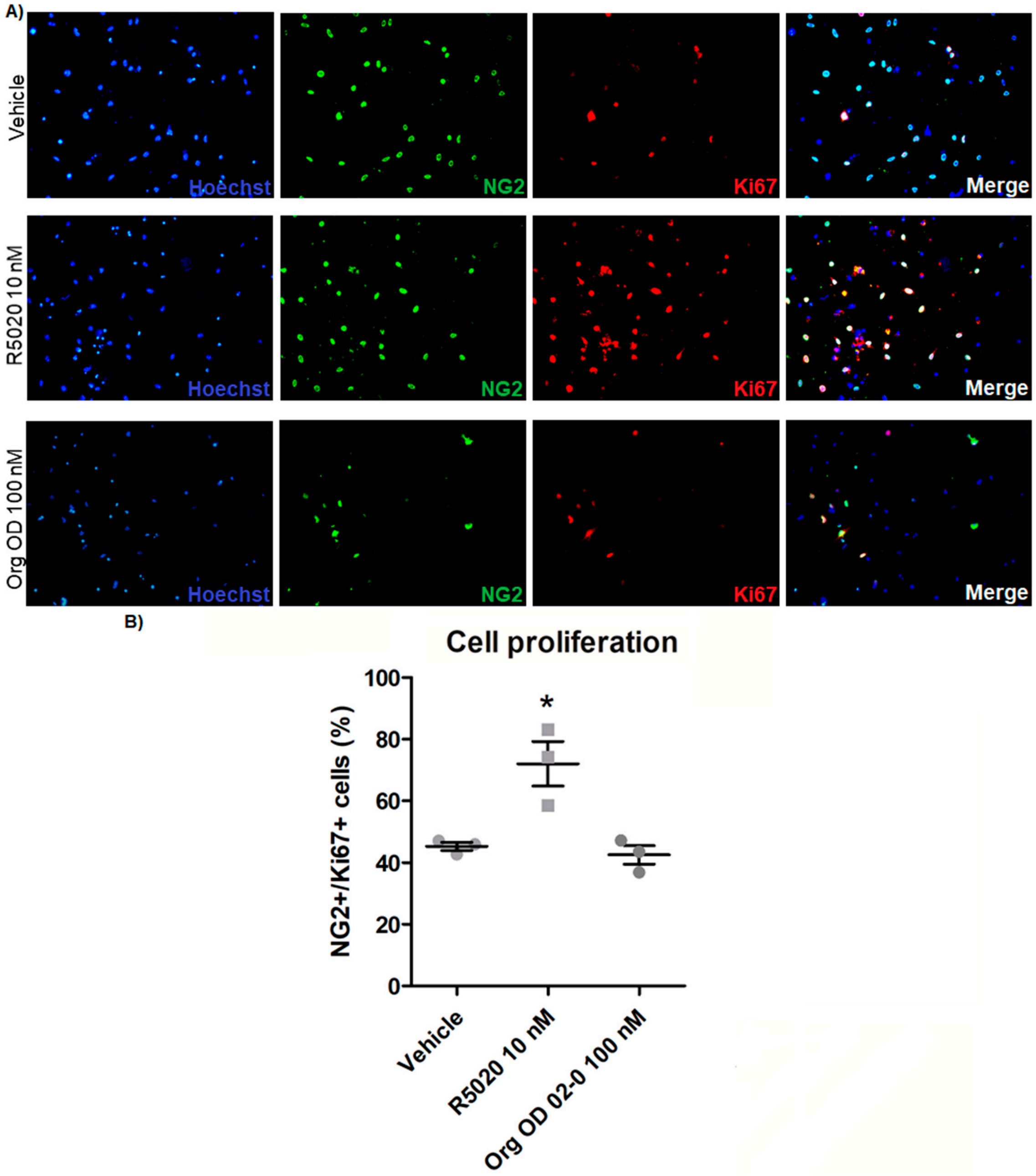

Figure S2. Progesterone increases embryonic OPC proliferation through PR. A) NG2/Ki67 immunostaining in OPC cultured with growth factors and treated for 3 days with the PR agonist R5020 (10 nM), the mPRs agonist Org OD 02-0 (100 nM) and vehicle (DMSO 0.01%). B) Graph derived from the percentage of NG2/Ki67 positive cells observed in the immunofluorescence experiments. Results are expressed as the mean ± S.E.M. *p<0.05 *vs* the rest of the groups.

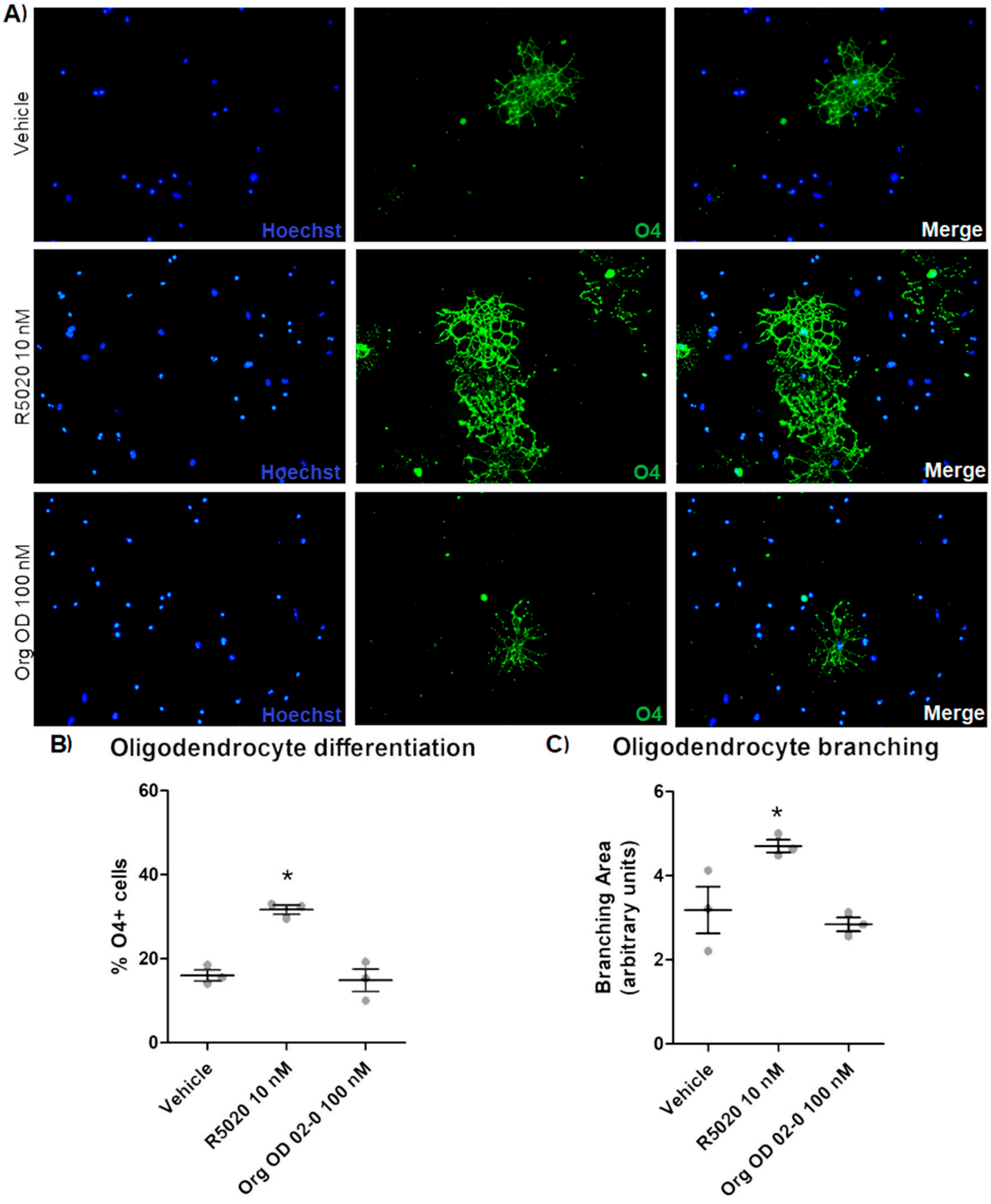

Figure S3. Progesterone promotes the oligodendrocyte differentiation through PR. A) O4 immunofluorescence in OPC cultured without growth factors and treated for 3 days with the PR agonist R5020 (10 nM), the mPRs agonist Org OD 02-0 (100 nM) and vehicle (DMSO 0.01%). B) Percentage of O4 positive cells observed in the immunofluorescence experiments. C) Cellular branching measured in O4 positive cells. Results are expressed as the mean ± S.E.M. *$p<0.05$ vs the rest of the groups.

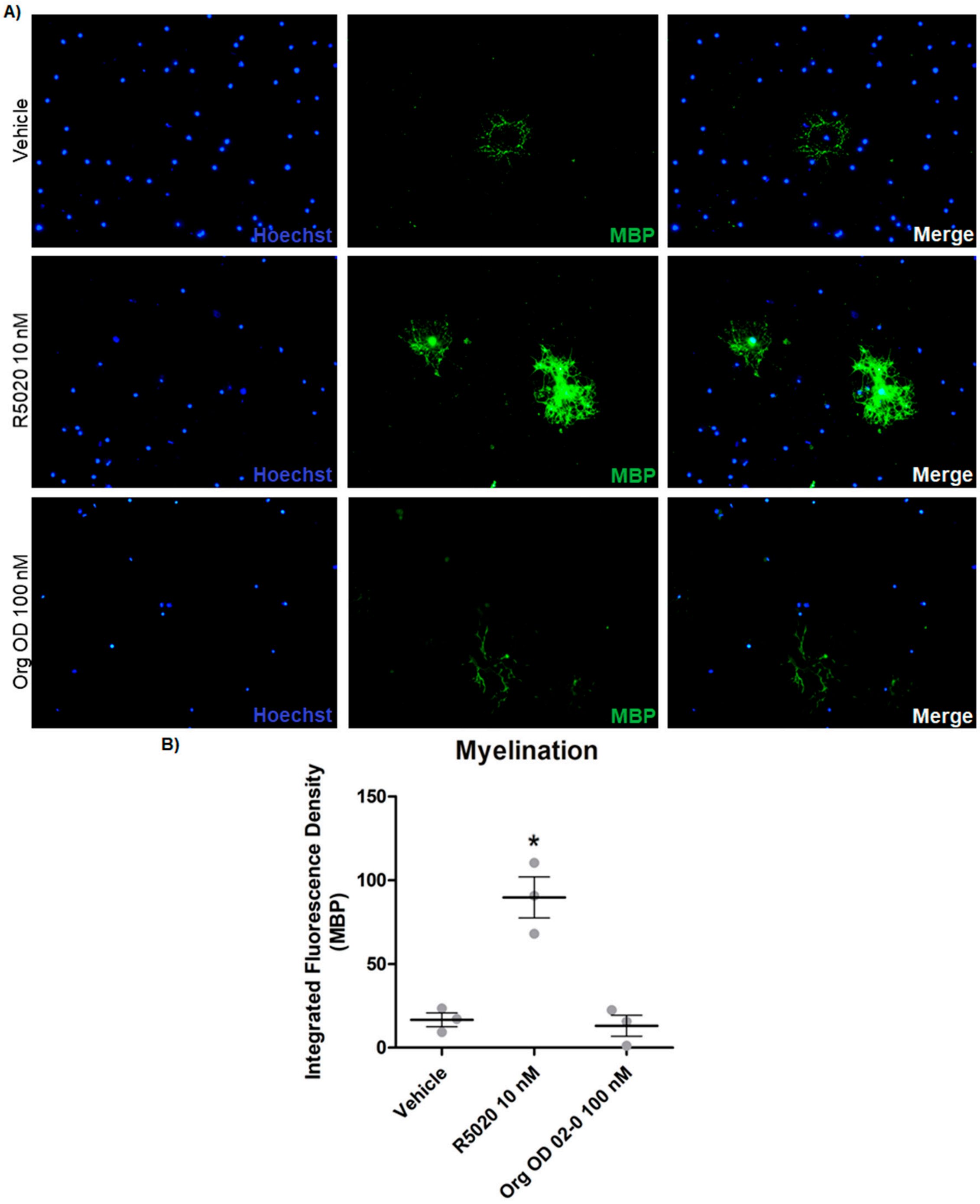

Figure S4. Progesterone increases the potential of myelination through PR. A) MBP immunofluorescence in OPC cultured without growth factors and treated for 3 days with the PR agonist R5020 (10 nM), the mPRs agonist Org OD 02-0 (100 nM) and vehicle (DMSO 0.01%). B) MBP expression measured as a fluorescence density. Results are expressed as the mean ± S.E.M. *$p<0.05$ vs the rest of the groups.

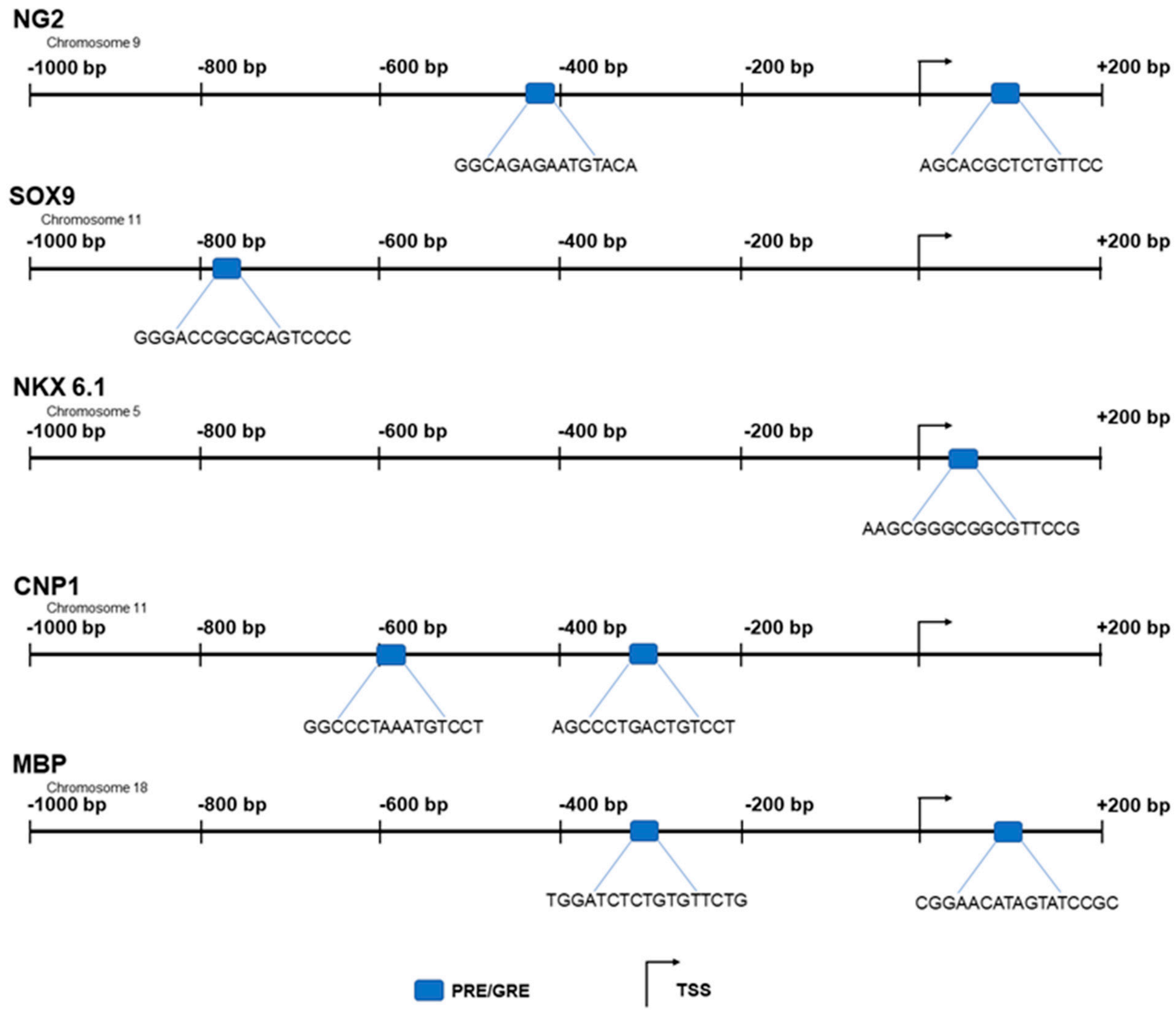

Figure S5. In silico analysis to identify potential PRE sites in the promoter sequences of NG2, SOX9, NKX 6.1, CNP1 and MBP genes. The promoter sequences were obtained from the Eukaryotic Promoter Database (EPD) and analyzed with the algorithms contained in the JASPAR, TRANSFAC and NUBIscan databases. For each sequence, black arrow indicates the transcription start site (TSS). The putative PRE are denoted with a blue square.
